# A New Family of Receptor Tyrosine Kinases with a Venus Flytrap Binding Domain in Insects and Other Invertebrates Activated by Aminoacids

**DOI:** 10.1371/journal.pone.0005651

**Published:** 2009-05-21

**Authors:** Arnaud Ahier, Philippe Rondard, Nadège Gouignard, Naji Khayath, Siluo Huang, Jacques Trolet, Daniel J. Donoghue, Monique Gauthier, Jean-Philippe Pin, Colette Dissous

**Affiliations:** 1 Inserm U547, Université de Lille 2, Institut Pasteur de Lille, Lille, France; 2 CNRS UMR5203, Institut de Génomique Fonctionnelle, Inserm U661, and Université Montpellier 1,2, Montpellier, France; 3 Centre de Recherches sur la Cognition Animale, CNRS UMR 5169, Université Paul Sabatier, Toulouse, France; 4 Department of Chemistry and Biochemistry and Moores UCSD Cancer Center, University of California San Diego, La Jolla, California, United States of America; INSERM U567, Institut Cochin, France

## Abstract

**Background:**

Tyrosine kinase receptors (RTKs) comprise a large family of membrane receptors that regulate various cellular processes in cell biology of diverse organisms. We previously described an atypical RTK in the platyhelminth parasite *Schistosoma mansoni*, composed of an extracellular Venus flytrap module (VFT) linked through a single transmembrane domain to an intracellular tyrosine kinase domain similar to that of the insulin receptor.

**Methods and Findings:**

Here we show that this receptor is a member of a new family of RTKs found in invertebrates, and particularly in insects. Sixteen new members of this family, named Venus Kinase Receptor (VKR), were identified in many insects. Structural and phylogenetic studies performed on VFT and TK domains showed that VKR sequences formed monophyletic groups, the VFT group being close to that of GABA_B_ receptors and the TK one being close to that of insulin receptors. We show that a recombinant VKR is able to autophosphorylate on tyrosine residues, and report that it can be activated by L-arginine. This is in agreement with the high degree of conservation of the alpha amino acid binding residues found in many amino acid binding VFTs. The presence of high levels of *vkr* transcripts in larval forms and in female gonads indicates a putative function of VKR in reproduction and/or development.

**Conclusion:**

The identification of RTKs specific for parasites and insect vectors raises new perspectives for the control of human parasitic and infectious diseases.

## Introduction

Receptor tyrosine kinases (RTKs) are single-pass membrane proteins constituted of an extracellular ligand-binding domain and an intracellular kinase domain [1]. RTK-mediated signals play key roles in the regulation of various cellular processes, such as the control of cell growth, differentiation, metabolism, and migration. In mammals, a large number of RTKs have been identified and classified into 20 subfamilies that mostly differ by the structural organization of their ligand recognition domain. These include, for examples, the epidermal growth factor receptors (EGFRs or ErbBs), the fibroblast growth factor receptors (FGFRs), the insulin and the insulin-like growth factor receptors (IR and IGFR), the platelet-derived growth factor receptors (PDGFRs), the vascular endothelial growth factor receptors (VEGFRs) and the proto-oncogene receptor tyrosine kinase ROS [2]. The RTK family is even more complex when considering invertebrate receptors. For example *Caenorhabditis elegans* genome contains 40 RTKs of which only 13 can be classified in 10 out of the 20 subfamilies of human RTKs [3]. In addition, several unusual RTKs have been described in *Hydra vulgaris*, such as Lemon which is a RTK characterized by the presence of extracellular immunoglobulin-like repeats [4], or Sweet Tooth with an extracellular portion containing four C-type lectin-like domains [5]. In all cases, these receptors function as oligomers (either constitutive oligomers or ligand-induced oligomers), the activation process involving a precise control of the relative position of the TK domains, leading to their auto-phosphorylation and then, their self activation [2].

**Figure 1:**
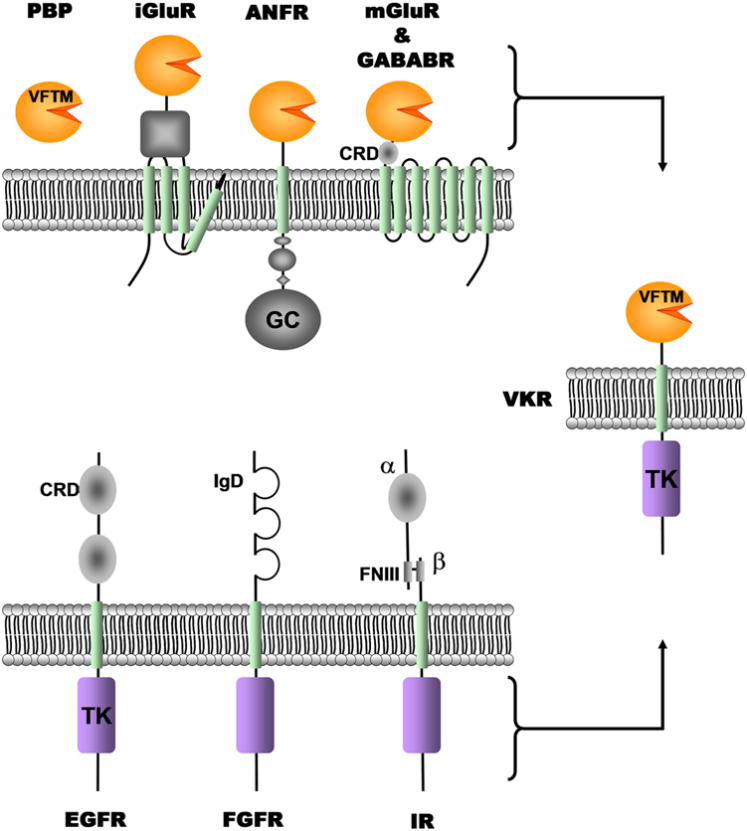
VKR are original proteins composed of a VFTM associated to a TK domain. The top panel represents the VFTM found in the bacterian periplasmic binding proteins (PBP) and in eukaryotic cell surface membrane receptors (iGluR: ionotropic Glutamate Receptor; ANFR: Atrial Natriuretic Factor Receptor; mGluR: metabotropic Glutamate Receptor; GABA_B_R: metabotropic α-aminobutyric acid Receptor). The bottom panel represents various RTK (EGFR: Epidermal Growth Factor Receptor; FGFR: Fibroblast Growth Factor Receptor; IR: Insulin Receptor composed of α and β chains). VFTM: Venus Flytrap Module, GC, Guanylate Cyclase domain; TK, Tyrosine Kinase domain; CRD: Cystein Rich Domain; IgD: Immunoglobulin Domain; FNIII: FibroNectin III Domain.

Recently, we reported the identification and characterization of an unexpected atypical RTK in the blood-dwelling fluke platyhelminth *Schistosoma mansoni*. This receptor is composed of an extracellular Venus Flytrap module (VFT), usually found in class C G-protein-coupled receptors such as the γ-aminobutyric acid type B (GABA_B_) receptor, linked through a single transmembrane domain to an intracellular tyrosine kinase similar to that of the IR [6]. This unusual receptor was called VKR for Venus Kinase Receptor. VFTs are large domains composed of two lobes connected via flexible tethers that close around the bound ligand similarly to the leaves of the Venus Flytrap carnivorous plant, *Dionaea muscipula*, when they catch their preys. VFTs were first identified as bacterial periplasmic-binding proteins (PBP) involved in the transport of small molecules, such as amino acids, sugars or ions [7]. VFTs constitute the binding pocket of different receptor types such as class C G-protein coupled receptors that are activated by amino acids (GABA or glutamate), sugar (the sweet taste receptor) or ions (the calcium sensing receptors), the ionotropic glutamate receptor iGluR, in which it constitutes a binding domain for natural allosteric modulators, and the atrial natriuretic peptide receptors (ANFR) with guanylate cyclase activity (Fig. 1) [8]. All these receptors function as homo- or hetero-oligomers, and the VFTs are directly involved in the oligomeric assembly. They even play an important role in receptor activation through the control of the general oligomeric organization of these receptors, mostly by controling the relative position of the protomers [9]–[12].

In this work, we characterized a new family of VKR proteins mainly present in insects, and showed that many of these receptors have specific expression patterns, being predominantly found in larvae and in the gonads of various organisms. Here, we provide direct evidence for their TK activity, their oligomeric assembly, and show that at least members of this new RTK family can be activated by amino acids such as L-arginine.

## Materials and Methods

### Biological material

Laboratory strains of *Apis mellifera*, *Tribolium castaneum* and *Drosophila pseudoobscura* were kindly provided respectively by Prof. Monique Gauthier, Prof. Eric Haubruge (FUSAGX, Gembloux, Belgique) and Prof. Geneviève Prevost (Université de Picardie, Amiens, France). *Anopheles gambiae* and *Strongylocentrotus purpuratus* dissected tissues were respectively furnished by Dr Catherine Bourgouin (CEPIA, Institut Pasteur, Paris, France) and Dr Karen Wilson (Kristineberg Marine Research Station, Fiskebäckskil, Sweden).

### *In silico* identification and gene structure analyses of VKR

tBLASTn searches were performed on different insect genomes available on FlyBase [version FB2009_01, released January 23, 2009] (flybase.bio.indiana.edu/) [13] and VectorBase [Last Updated January 23rd, 2009] (www.vectorbase.org) [14]. A two-step screening strategy was employed. First, *smvkr* gene was used as probe to search for similar genes in insect genomes. Sequences of candidate VKR genes were identified manually by analysing genomic DNA flanking open reading frames, using splice donor/acceptor consensus sequences (GT/AG) and the eukaryotic genomic gene prediction programs Augustus (http://augustus.gobics.de/) [15] and GENSCAN (http://genes.mit.edu/GENSCAN.html). Next, we ran secondary tBLASTn searches to identify additional insect VKR genes using the candidate insect VKR genes identified in the first step to increase accuracy of research. Additionally, the sea urchin VKR was found in the sea urchin genome database (http://urchin.nidcr.nih.gov/blast/index.html).

All the conserved exon-intron boundaries were identified by amino acid sequence alignments in a pair-wise manner using CLUSTALW [16] in BioEdit Sequenced Alignment Editor version 7.0.9.0 and revised by eye. This exon-intron structure was confirmed by the cDNA sequence when available. The domain organization of the putative proteins was deduced using algorithms from SMART “Simple Modular Architecture Research Tool” (http://smart.embl-heidelberg.de/). Transmembrane regions of VKR were confirmed by domain by Kyte and Doolittle Hydropathy Plot (http://gcat.davidson.edu/rakarnik/kyte-doolittle.htm) [17] and TMHMM server (http://www.cbs.dtu.dk/services/TMHMM/) [18].

### Comparative analyses of amino acid sequences and phylogenetic analysis

VFTM and TK domains were delimited by SMART and/or BLAST structural analysis algorithms. Pair-wise mannered alignments were generated with Clustal W program (MEGA 4) [19] and slightly modified by eye. Phylogenetic reconstruction was performed using the MEGA 4 neighbour joining method, based on a Poisson correction substitution model, to generate unrooted best phylogenetic tree. Bootstrapping was performed to estimate the confidence of the branches using 10 000 neighbour joining replicates [20]. Genbank accession numbers of receptors used are in table S1.

### cDNA cloning and sequencing of insect VKRs

Total RNA was extracted from whole adult insect tissues using Nucleospin RNA II kit according to the manufacturer's procedure (Clontech Laboratories, Inc.). Reverse transcription was performed for 1 h at 55°C in a total volume of 25 µl with 1 µg total RNA per sample following the Thermoscript standard procedure (Invitrogen, Inc). Dpseuvkr, Agvkr, Tcvkr and Amvkr cDNAs were amplified by PCR using Platinum Hi-Fi Taq DNA polymerase (Invitrogen) and the following primer sequences respectively ( Dpseuvkr R 5′-GAGTGGGGACATTATACTGTATCCATCTGGGGT-3′, Dpseuvkr F 5′-GCGCTCAGGTATATAGAAATCCGAACTGCG-3′, Agvkr R 5′-CGGACACCTCCGAGGCGGAGGGT-3′, Agvkr F 5′-GCGTGAAGTGTGCGCTGGTGCTGG-3′, Tcvkr R 5′-AGCGGCCGCGACATTGG AGACAACGTAGCCATTC-3′, Tcvkr F 5′-AGGATCCAGGCATCGTCCACGGGGC-3′, Amvkr R 5′-GCTATCTAGAAAGAATCGACACGTCCTCCGACTTTC-3′ and Amvkr F 5′-GGACCGGTAAATCGTGATCTCAAATCGATGTCTG-3′). PCR products were purified from agarose gels using the gel extraction kit JETSORB (Genomed, Inc) and inserted into pCR2.1-TOPO (Invitrogen). AmVKR cDNA sequence was subcloned into the prk5-ST-HA mammalian expression vector containing the human mGluR5 signal sequence, SNAP and HA epitope [21] by an in-frame insertion using Age1 and Xba1 sites. Selected clones of AmVKR constructs were sequenced by Genoscreen (Lille, France). Sequences were reviewed by visual inspection with Chromas software (version 1.45; Technelysium).

### Quantitative RT-PCR

*vkr* transcripts were quantified in different developmental stages or tissue types of the organisms using the technique of real-time RT-PCR. Total RNA was extracted using Nucleospin RNA II kit according to the manufacturer's procedure (Clontech Laboratories, Inc.) and reverse transcribed using the Thermoscript™ RT-PCR System (Invitrogen). cDNAs were used as templates for PCR amplification using the qPCR Mastermix SYBR Green I sequence detection system (Eurogentec). VKR specific and internal standard primers were designed by the primer express program (Applied Biosystems) and used for amplification in triplicate assays.

*Agvkr* (GenBankTM accession number EU878397, positions 3386–3404; 3417–3436)

*Agactin* (GenBankTM accession number CR954256, positions 900–919; 931–950)

*Spvkr* (GenBankTM accession number BK006716, positions 3568–3587; 3610–3630)

*Sptubulin* (GenBankTM accession number XM_001177294.1, positions 599–618; 630–549)

*Tcvkr* (GenBankTM accession number EU878395, positions 2154–2170; 2184–2205)

*Tctubulin* (GenBankTM accession number XM_961491.1, positions 687–706; 718–737)

*Amvkr* (GenBankTM accession number EU878396, positions 27–49; 105–127)

*Amactin* (GenBankTM accession number XM_623378.1, positions 1325–1344; 1405–1425)

For graphical representation of quantitative PCR data, the delta-delta Ct (ΔΔCt) method [22] was applied.

### Site-directed mutagenesis

A dead-kinase AmVKR (AmVKR^dk^) construct was obtained by site-directed mutagenesis of the active D_1031_FG_1033_ motif into a D*NA* inactive motif using the QuickChange®Site-Directed Mutagenesis Kit (Stratagene) with the 5′-GTGAAGCTTGGAGAC*aa*TG*c*TATGACGAGGTTG-3′ mutated sequence and its reverse complement (mutated residues are in lowercase italic). Other mutations concerned the K_1043_ and F_1044_ residues next to the potential YY_1042_ autophosphorylation site of AmVKR by using respectively the 5′-CGAGAACGATTACTAC*g*AGTTCAATCGAAGAGGTATG-3′ (AmVKR^YYEF^) and the 5′-GTACGAGAACGATTACTACAAG*gag*AATCGAAGAGGTATGC-3′ (AmVKR^YYKE^) mutated sequences and their reverse complement. The AmVKR^YYEE^ construct with a double mutation on K_1043_ and F_1044_ residues was obtained by mutating a selected clone of AmVKR^YYEF^ with the 5′-CGAGAACGATTACTACGAG*gag*AATCGAAGAGGTATG-3′ primer.

### Cell transfection procedures

Human embryonic kidney 293 (HEK293) cells were cultured in DMEM-Glutamax (Gibco-BRL) supplemented with 10% fetal calf serum (FCS). Cells were grown in an incubator at 37°C with humidified 5% CO_2_ atmosphere. 4×10^5^ cells were cultured in 6-well plates and transiently transfected using a polyethylenimine (PEI) method in the presence of 10% FCS according to the manufacturer's instructions. Cells were incubated for 24 h in 1 ml of culture medium with 1 µg of AmVKR or various mutant plasmid DNA and 3,3 µl of Exgen500 (Fermentas). Recombinant protein expression was detected by Western blot of transfected cell lysates or by immunocytochemistry on fixed (not permeabilized) cells using HA-Tag (6E2) mouse monoclonal antibodies according to the manufacturer's instructions (Cell Signaling).

### In vitro kinase assays

Kinase activity of native or mutated forms of recombinant AmVKR was detected on proteins immunoprecipitated from detergent lysates of HEK 293 cells transfected by AmVKR constructs for a period of 24 h in the conditions described above. Briefly, 10^6^ cells were washed twice in PBS pH 7.4, then lysed in 500 µl lysis buffer (50 mM Tris pH 7.8, 150 mM NaCl, 1% Nonidet P40), placed on ice for 15 min and centrifuged 30 min at 10 000 g. Cell lysates were added with HA-Tag (6E2) antibodies (1∶200 final dilution) and incubated at 4°C overnight together with 5 mg protein G- agarose beads (Sigma). Beads were washed three times with lysis buffer, twice with kinase buffer (50 mM HEPES, pH 7.5, 12.5 mM MgCl_2_, 150 mM NaCl, 1 mM dithiothreitol, 1 mM Na_3_VO_4_) containing protease inhibitor cocktail (Sigma, 1∶1000 final), then incubated in a total volume of 20 µl kinase buffer in the presence of 50 µM ATP at 30°C for 30 min. Kinase reactions were stopped by the addition of 6,6 µl of 4× SDS–PAGE sample buffer and heated at 70°C for 3 min. Eluates were analysed by SDS-PAGE in a 8% polyacrylamide gel, blotted onto nitrocellulose membrane and proteins were detected by Western blot using HA-Tag (6E2) (1∶5000) or P-Tyr-100 (1∶2000, Cell Signaling) antibodies. Anti-mouse rabbit antibodies conjugated to horseradish peroxidase (Sigma) were used as secondary antibodies and signals were detected using the SuperSignal West Femto™ ECL kit (Pierce).

### SNAP-tag labeling and time resolved-FRET SNAP-tag measurements with compatible fluorophores

Twenty-four hours after transfection with AmVKR constructs (prk5-ST-HA plasmids), HEK293 cells (100,000 per well of a Greiner CellStar 96-well plate) were washed with DMEM and 10% fetal calf serum (FCS) and labeled with BG (Benzyl Guanine) conjugated with fluorophore europium cryptate (BG-K) at 4 µM or acceptor (BG-d2) at 0.5 µM for 1 h at 37°C, in 5% CO_2_ in DMEM 10% FCS, as previously described [21]. Cells were washed four times with Tris-KREBS and we measured the FRET signal at 665 nm with a 50 µs delay after laser excitation at 337 nm using a Rubystar plate reader (BMG Labtechnologies). We calculated the FRET intensity as (total signal at 665 nm) – (background at 665 nm), where the background signal corresponds to cells labeled with the donor fluorophore alone. Untransfected HEK cells (mock cells) were used as control.

### TK and VFTM modelling

A homology model of the AmVKR VFTM of VKR was generated using the crystal structure of mGlu1 VFT (PDB accession number 1EWK) as a template. Models were manually refined with ViTO [23] using the sequence alignment of AmVKR, AgVKR, TcVKR, SpVKR and SmVKR. Final models were built using Modeller 7.0 [24] and evaluated using the dynamic evolutionary trace as implemented in ViTO.

An evolutionary trace analysis of TK domain and VFTM of VKR was performed after aligning sequences of each domain, including those from insects and other invertebrates (*S. mansoni* and *S. purpuratus*). The alignment generated with ClustalW was submitted to the ConSurf website server (http://Consurf.tau.ac.il) [25]. Conservation scores of each residue were calculated by taking into account the phylogenetic relationships among the sequences and the similarity between the amino acids in the alignment.

## Results

### Identification of a *vkr* gene family in invertebrates

**Figure 2:**
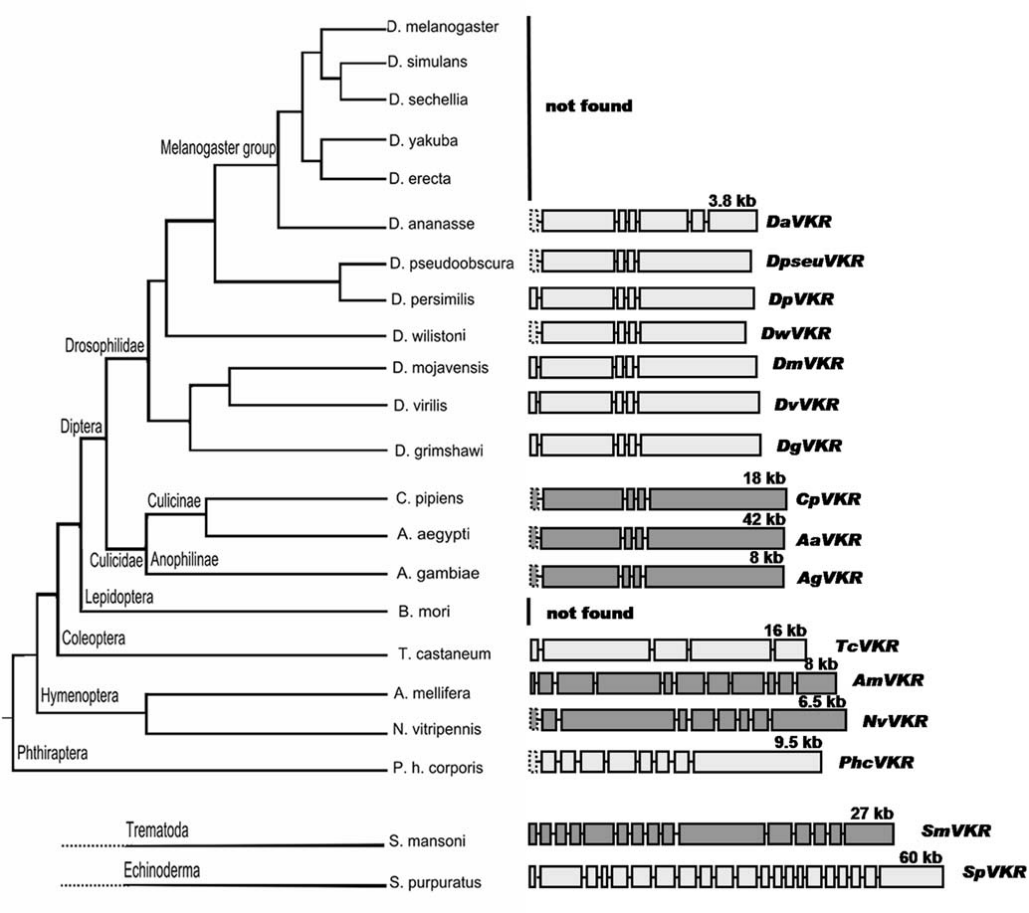
Genomic structure of vkr genes. Genes were ordered following the FlyBase hierarchical tree that showed insect species for which data were available. Predicted exons were represented by rectangles drawn to scale for each vkr gene. Genes of seven Drosophila species (*D. ananasse*, *D. pseudoobscura*, *D. persimilis*, *D. wilistoni*, *D. mojavensis*, *D. virilis*, *and D. grimshawi*), three mosquitoes (*C. pipiens*, *A. aegypti*, *A. gambiae*), the coleopteran T. castaneum, two hymenopteran species (*A. mellifera and N. vitripennis*), the phthirapteran *P. humanus corporis*, the trematode *S. mansoni* and the echinoderm *S. purpuratus* are presented. Numbers indicate respective gene lengths in kilobases. Not found indicates that the presence of the vkr gene was not found in the species studied.

In order to examine whether SmVKR [6], the atypical RTK identified in *S. mansoni* was exclusively present in this organism, or was common to other species, we searched for possible orthologs in other animal genomes. A recursive search strategy starting with the SmVKR sequence and using automatic ortholog annotation in combination with the TBLASTN algorithm, allowed us to discover fourteen novel *vkr* genes in various insect genomes (Fig. 2). Putative *vkr* orthologs were identified in several Diptera including *Drosophila* (*D. ananasse* (Genbank Accession no. BK006724), *D. pseudoobscura*, *D. persimilis* (BK006723), *D. willistoni* (BK006722), *D. mojavensis* (BK006720), *D. virilis* (BK006719) *and D. grimshawi* (BK006721)) and in mosquito (*Anopheles gambiae*, *Aedes aegypti* (BK006725) and *Culex pipiens* (BK006726)) species, as well as in Coleoptera (*Tribolium castaneum*), Hymenoptera (*Apis mellifera* and *Nasonia vitripennis* (BK006718)) and Phthiraptera (*Pediculus humanus corporis* (BK006717)) organisms. Surprisingly, whereas *vkr* genes were present in the genome of several dipteras, including seven drosophilidae, *vkr* was not found in the genome of any species belonging to the *melanogaster* group (Fig. 2). Similarly, *vkr* genes were not detected in any vertebrate genome, nor in the genome of the nematode *Caenorhabditis elegans*. However, a *vkr* sequence was discovered in the sea urchin *Strongylocentrotus purpuratus* [26], indicating that *vkr* genes were not specific to protostomia, but can also be found in deuterostomia.

**Figure 3:**
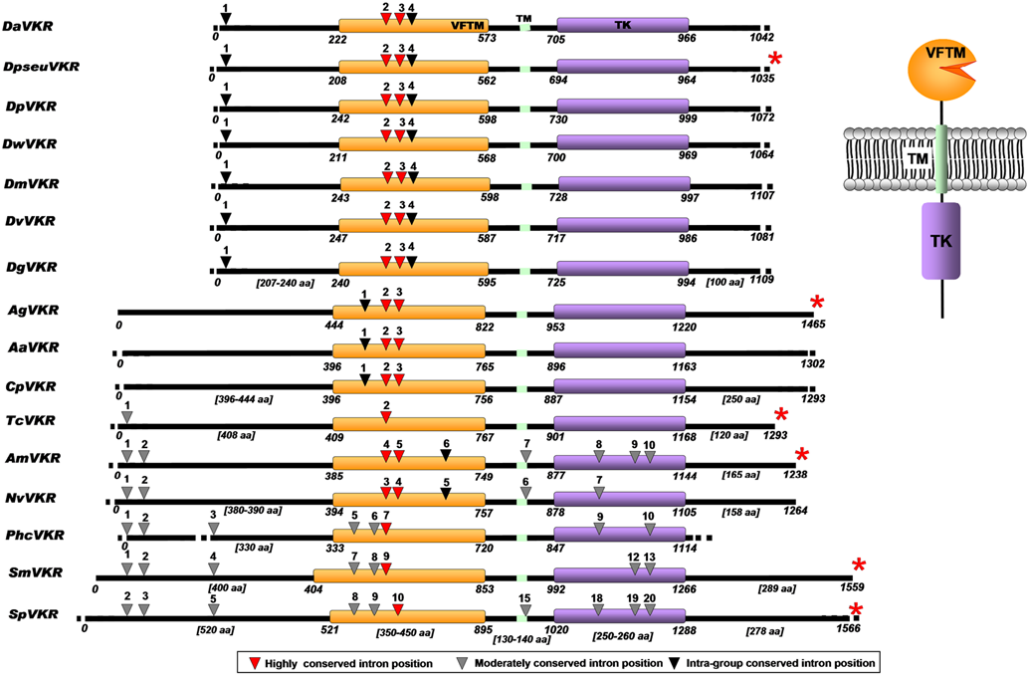
Conserved structure of receptors composing the VKR family. VFTM, TM and TK domains are respectively shown in orange, green and purple. Arrows give the positions of conserved introns in the different sequences with their corresponding numbers in each sequence. The different colors used for arrows indicated variable degrees of conservation for intron positions, as mentioned. The positions of residues delimiting the different domains are indicated by numbers. Red asterisks correspond to VKR for which cDNAs were cloned and sequenced.

*Vkr* genes were shown to be highly variable in size. *Vkr* genes of drosophilidae ranged from 4 to 5,000 bp whereas those of mosquitoes measured up to 40,000 bp. In other species, *vkr* genes ranged from 6 to 25,000 bp, with the exception of *S. purpuratus* in which the size of the gene was remarkably larger (about 60,000 bp). *Vkr* genes also showed a variable multi-exon structure in their coding region. Mosquito and fly genes contained a relatively small number of exons (varying from 4 to 6) whereas those of *S. mansoni* and *S. purpuratus* were shown to be composed of 15 and 21 exons respectively. Intron sizes were highly heterogeneous and ranged in size from about 10 to 30,000 bp (as for the terminal intron of *Aavkr*). In spite of their variable complexity and their structural differences, most of the *vkr* genes were shown to contain a large C terminal exon coding for half of the VFTM, the TM hinge region and the entire intracellular part of the protein including the TK catalytic domain (Fig 3). This observation was sufficient to rule out the possibility that VFTM and TK sequences would have been incorrectly fused during contig analysis and cDNA sequence assemblies and therefore indicated that a new family of RTK proteins exists associating VFT and TK domains. To confirm these data obtained from *in silico* sequence analysis we cloned and sequenced the *vkr* transcripts amplified from various insect species like the adult fly of *D. pseudoobscura* (EU598264) (Diptera), the mosquito *A. gambiae* (EU878397) (Diptera), the red flour beetle *T. castaneum* (EU878395) (Coleoptera) and the honey bee *A. mellifera* (EU878396) (Hymenoptera).

### Phylogenetic analysis and structural conservation of VKR proteins

Sequencing of the different cloned *vkr* cDNAs allowed us to confirm that VFT and TK domains were effectively encoded by a single transcript, as it was already demonstrated for *Smvkr*. Further analysis of full-length cDNA and deduced protein sequences indicated that VFT and TK domains were separated by an hydrophobic region rich in isoleucine, leucine and valine, and identified as the transmembrane (TM) domain by Kyte and Doolittle Hydropathy Plot and TMHMM analysis (with a probability value of 1). No other known protein family domain could be revealed in the various VKR structures.

**Figure 4:**
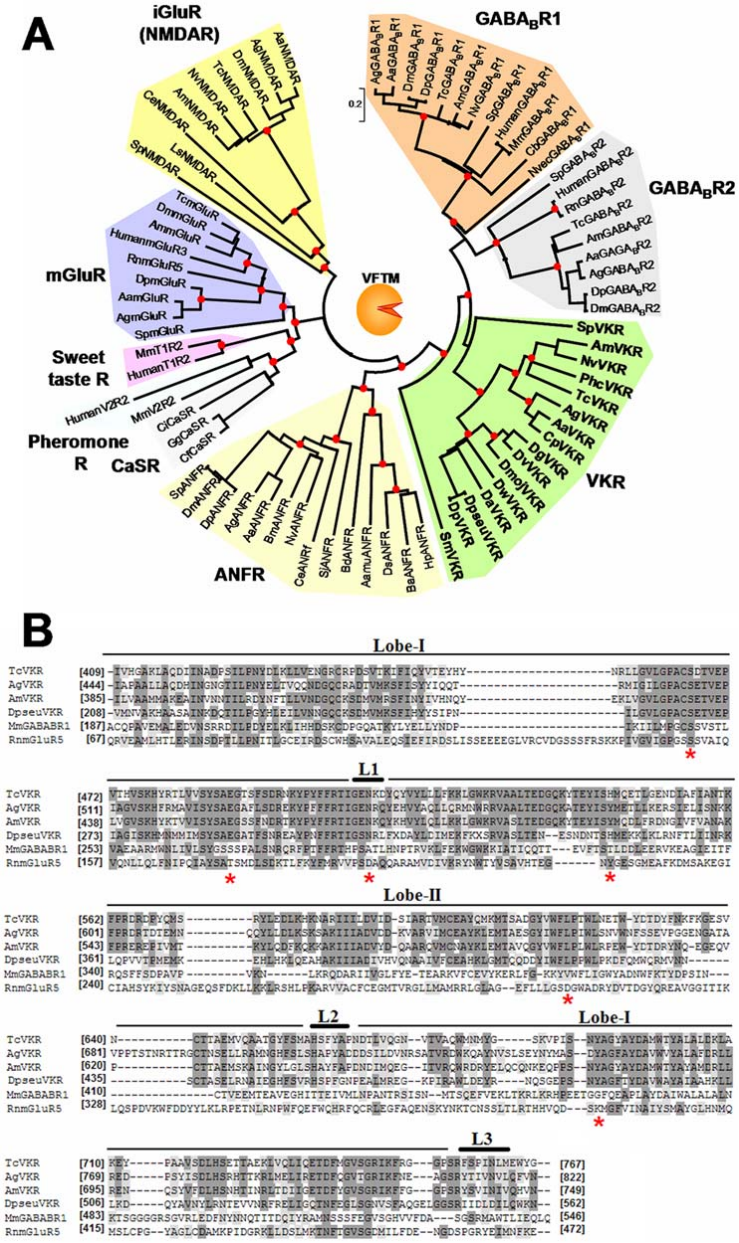
Phylogenetic analyses and sequence alignment of VFTM from VKR. A-Phylogenetic relationship of VFT protein domains from VKR and VFT-containing receptors, GABA_B_ receptor (GABA_B_R1 and GABA_B_R2 subunits), the natriuretic peptide receptor (ANFR), the metabotropic glutamate-like receptors (mGluR, sweet taste, pheromone and CaSR calcium receptors) and the iGluR N-methyl-D-aspartate (NMDA) receptor mostly from insects and invertebrates. Bootstrap values (10 000 replicates) higher than 80% were shown by a red point on the major internal node only. Genbank accession numbers of receptors used are in table S1. B- Sequence alignment of VFT protein domains from different insect VKRs (AmVKR, TcVKR, AgVKR and DpseuVKR for which cDNA was cloned and sequenced) with the extracellular domain of the human GABABR1 (Q9UBS5) and of the *Rattus Norvegicus* (NP_058708.1) mGluR5 subunit using the CLUSTAL W method. Residues highlighted in black are those identical in at least 50% of the sequences. Those in grey background correspond to residues homologous in at least 50% of the sequences. The positions highlighted in red with an asterisk are those important for glutamate binding in the mGluR5 subunit.

VFTM sequences of VKR proteins were compared to those of known VFTM-containing receptors including class C GPCRs (mGluR, GABA_B_R1/2, pheromone, sweet and umami taste and CaS receptors), receptors with guanylate cyclase activity (ANFR) and NMDA ionotropic glutamate receptor (iGluR). With the exception of pheromone and sweet taste receptors which are found only in high vertebrates [27], all of these VFTM-containing receptors have been shown to be present both in invertebrates (from cnidaria to insects) and vertebrates. Phylogenetic data obtained from an alignment implying predominantly insect VFTM sequences, showed that all VKR proteins effectively formed a distinct monophyletical protein clade (about 80% of identity between VKR of insects) which is closely related to GABA_B_R (with a 92% bootstrap score)( Fig 4A). Since the VKR class apparently arose before the divergence of GABA_B_R1 and GABA_B_R2 sub-classes, it was not possible to determine whether VKRs were closer to one than to the other receptor subclass. Inside of the VKR group, overall sequences were ordered according to an expected phylogeny, respecting the subgroups of dipteran (flies and mosquitoes) and hymenopteran lineages and thus reinforcing relationships between VKR sequences.

According to the evolutionary analysis, sequence alignment (Fig. 4B) indicated that VFTM of VKR proteins exhibit all the characteristics of GABA_B_R1 homologs with the two lobes I and II, composing the ligand binding domain, as well as the linkers, L1, L2 and L3 which interconnect both lobes.

**Figure 5:**
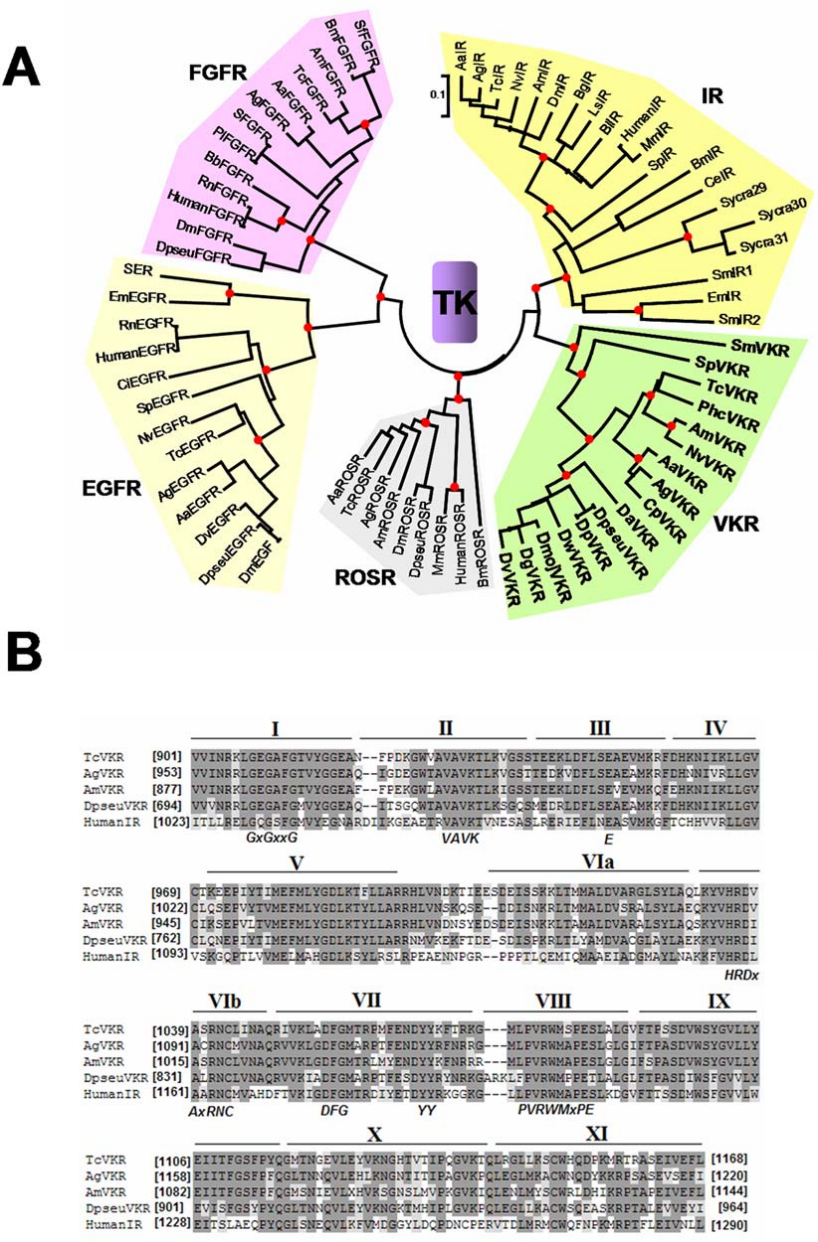
Phylogenetic analyses and sequence alignment of TK domains from VKR. A-Phylogenetic relationships of TK from VKR and various RTK, the receptors for insulin (IR), the epidermal growth factor (EGFR), the fibroblast growth factor (FGFR) and the proto-oncogene c-ros receptor (ROSR). Bootstrap values (10 000 replicates) higher than 80% were shown by a red point on the major internal node only. Genbank accession numbers of receptors used are in table S1. B- Sequence alignment of the catalytic domains from insect VKR (AmVKR, TcVKR, AgVKR and DpseuVKR) with the TK domain of human insulin receptor (NP_000199) using the CLUSTAL W method. Shaded areas represent residues which are identical (in black) or similar (in grey) in at least 50% of the aligned sequences. Numbers I to XI indicate the eleven subdomains conserved in kinase domains. Consensus sequences required for TK activity are in bold italics.

Similarly, phylogenetic relationships between the TK domains from VKRs and from various RTKs (IR, EGFR, FGFR, and ROS) showed that all VKR TK domains formed a monophyletic group close to TK domains of IRs (with a 80% bootstrap score) (Fig. 5A). Moreover, as obtained for VFTM, the TK domains of VKRs appeared to be ordered according to an expected phylogeny, sharing between them high percentages (50 to 80%) of amino acid identity, probably as the result of the large and general conservation of TK catalytic domains.

VKR TK domains are divided into eleven subdomains, as expected for RTKs [28] (Fig. 5B). All the motifs crucial for tyrosine kinase activity such as the ATP binding site (***GXGXXG***), the sequence required for ATP stabilization (***VAVKX_16_E***), the catalytic loop implicated in phosphotransfer (***HRDXAXRN***), the Mg^2+^ binding site (***DFG***), the consensus ***PVRWMXPE*** sequence considered as a strong indicator of tyrosine substrate specificity and the two putative juxtaposed autophosphorylation sites (***YY***) found in a limited number of RTK, including IR [28], are present in VKR (Fig. 5B). Surprisingly, no NPXY binding motif for IRS (Insulin Receptor Substrate) which is essential for signal transduction of IR members could be detected in any VKR sequences, nor SH2 (Src-homology 2) binding motifs (YXXM) described in most of the TK proteins.

All together, these results demonstrate that VKRs are formed by the original association of two already-known domains which are the GABA_B_R VFTM for the extracellular part of the receptor and an IR-like TK domain for its intracellular part.

### VKRs are expressed in larvae, and in gonads of adults

**Figure 6:**
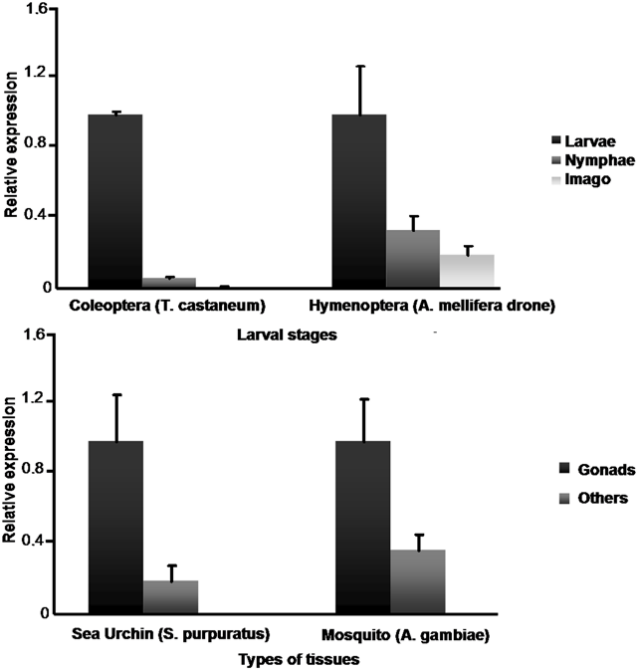
VKR are expressed in larvae and in gonads of adult insects and invertebrates Vkr transcripts were quantified in the different developmental stages of *T. castaneum* and of *A. mellifera* drone (A) and in tissues of *A. gambiae* and *S. purpuratus* (B). For graphical representation of qPCR data, cycle thresholds (Δ Ct values) obtained for the different samples were deducted from the Δ Ct value obtained for larval stage (A) or gonad (B) transcript levels. Values were normalized as relative fold-difference using the Δ-Δ Ct (ΔΔ Ct) method. Data are means±s.e. of triplicates from a typical experiment.

We investigated the expression of *vkr* genes in different insects and analysed the relative amount of transcripts in various developmental stages or tissue types by real-time RT-PCR. In the insects *T. castaneum* and *A. mellifera* drone, we observed that *vkr* transcripts were much more abundant in larval stages than in others like nympha and imago (Fig 6A). Further experiments also indicated that transcription of *vkr* genes was particularly active in gonad tissues of sea urchin *S. purpuratus* and mosquito *A. gambiae* in which we could detect respectively 5 and 2.5 fold more transcripts in female genital organs than in the rest of the body (Fig 6B). These results could argue for a role of VKR proteins in embryonic and larval development (as in *T. castaneum* and *A. mellifera*) as well as in genital organs and reproductive activity (in *A. gambiae* and *S. purpuratus*).

### VKR are functional tyrosine kinase receptors

**Figure 7:**
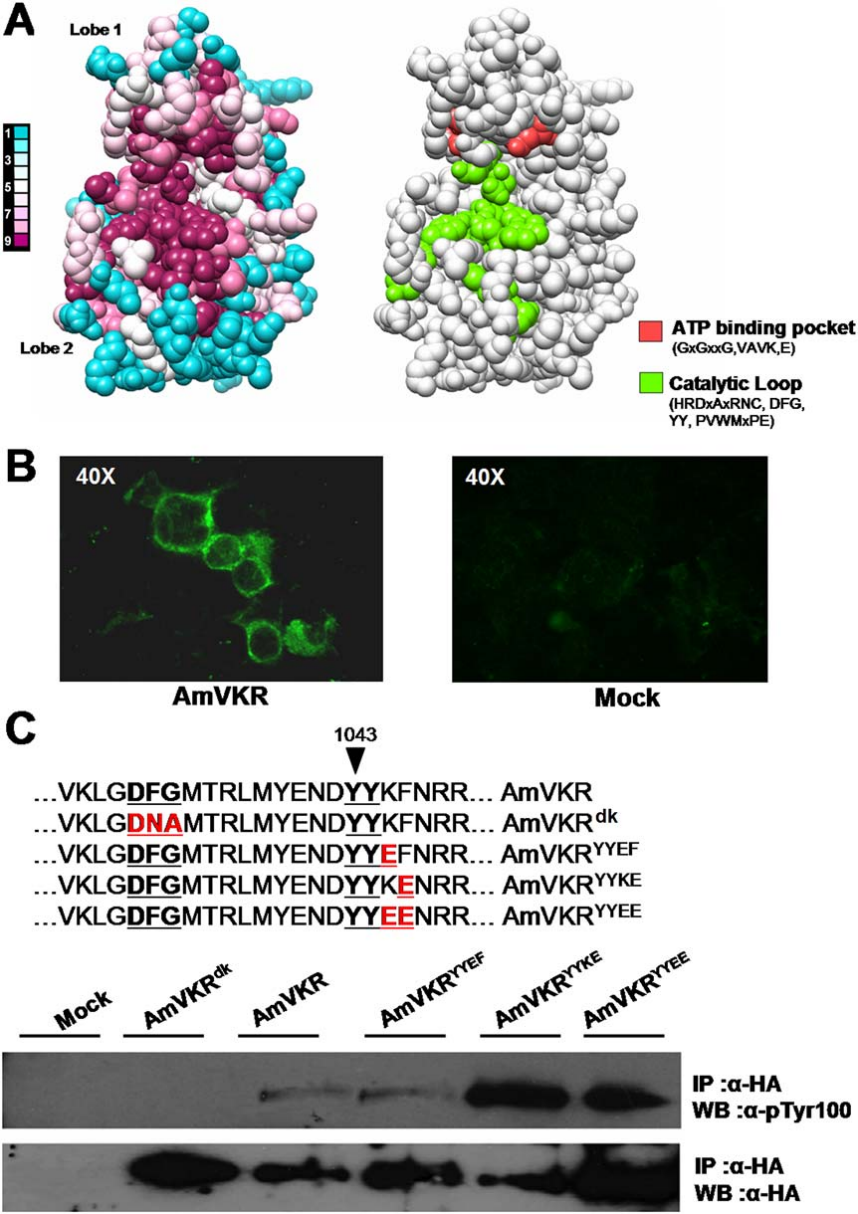
AmVKR shows tyrosine kinase activity. A- Evolutionary conservation of residues of the VKR TK domain (left panel) visualized on the human IR TK crystal structure (PDB accession number 1IR3). Conservation scores are according to a color scale from variable (blue) to conserved (purple) residues. For comparison, the crucial residues needed for kinase activity (right panel) are indicated. B- Expression of the HA-tagged AmVKR in HEK-293 transfected cells revealed by anti-HA antibodies. C- Tyrosine kinase activity of HA-tagged AmVKR proteins. Lysates from HEK293 cells transfected by plasmids containing AmVKR or mutated versions of AmVKR, or by empty prk5 plasmid as a control, were immunoprecipitated by anti-HA antibodies. Kinase assays were performed as described in Materials and Methods. Proteins were analysed by Western blot and tyrosine phosphorylation was detected using P-Tyr-100 antibodies.

To predict whether the VKR proteins retained a kinase activity, we first analysed the conservation of residues of the TK domain using a sequence alignment of the TK domain of the 16 VKRs and the crystal structure of the TK domain of the human insulin receptor (PDB accession number 1irk; [29]). This evolutionary trace analysis revealed that residues composing the catalytic loop and the ATP-binding site of kinase domains were highly conserved and identical among all VKRs (Fig. 7A).

Experimental studies about the functional activity of VKR proteins have been performed essentially on AmVKR, the receptor of honey bee. Similarly to different insect receptors which were already shown to be expressed with efficacy in epithelial mammalian cells (for example *A. mellifera* [30] and *D. melanogaster* [31] mGluR), we demonstrated that HEK cells transfected by AmVKR expressed significant amounts of the protein. Moreover, it was observed that the insertion of human mGluR5 signal peptide sequence upstream of the AmVKR sequence optimized significatively the expression of the recombinant protein at the surface of the cells. The substantial labeling of non-permeabilized transfected cells by anti-HA antibodies (directed against the N terminal tag) demonstrated the presence at the cell surface of AmVKR as well as its correct position in the membrane (Fig 7B). The presence of AmVKR in transfected cells was further confirmed by Western blot analysis of cell lysates (results not shown) and of proteins immunoprecipitated from cell lysates by anti-HA antibodies. Results showed that a 170 kDa band was consistently and specifically recognized blots by anti-HA antibodies (Fig. 7C).

In order to analyse kinase activity of recombinant AmVKR, we developed an *in vitro* kinase assay in which we measured the ability of immunoprecipitated VKR to autophosphorylate in kinase buffered conditions. Results in Fig. 7C indicated that AmVKR was autophosphorylated at a low level, similarly to various RTKs in the absence of ligand activation. To verify the specificity of kinase reactions, we used a dead-kinase mutant AmVKR^dk^ in which the D_1031_FG_1033_ Mg^2+^ binding motif essential for kinase activity was mutated into a D*NA* inactive motif. Then, AmVKR kinase potential was further confirmed using mutated versions of AmVKR in which glutamic residues were placed near the YY activation site, thus potentially mimicking tyrosine phosphorylation and receptor activation, such as the activating mutation found in fibroblast growth factor receptor 3 (FGFR3) in the type II neonatal thanatophoric dysplasia disease [32]. Three different mutants AmVKR^YYEF^, AmVKR^YYKE^ and AmVKR^YYEE^ were thus prepared by replacing in AmVKR, either K_1045_ or F_1046_ or both K_1045_/F_1046_ by E residues. When tested in the *in vitro* kinase assay, AmVKR^YYKE^ and AmVKR^YYEE^ mutants displayed strong kinase activity as compared to the wild-type AmVKR or to AmVKR^YYEF^. These results indicated the importance of the conserved YY site for activity of VKR proteins and confirmed the potential activity of their TK domain.

### VKR can form dimers

**Figure 8:**
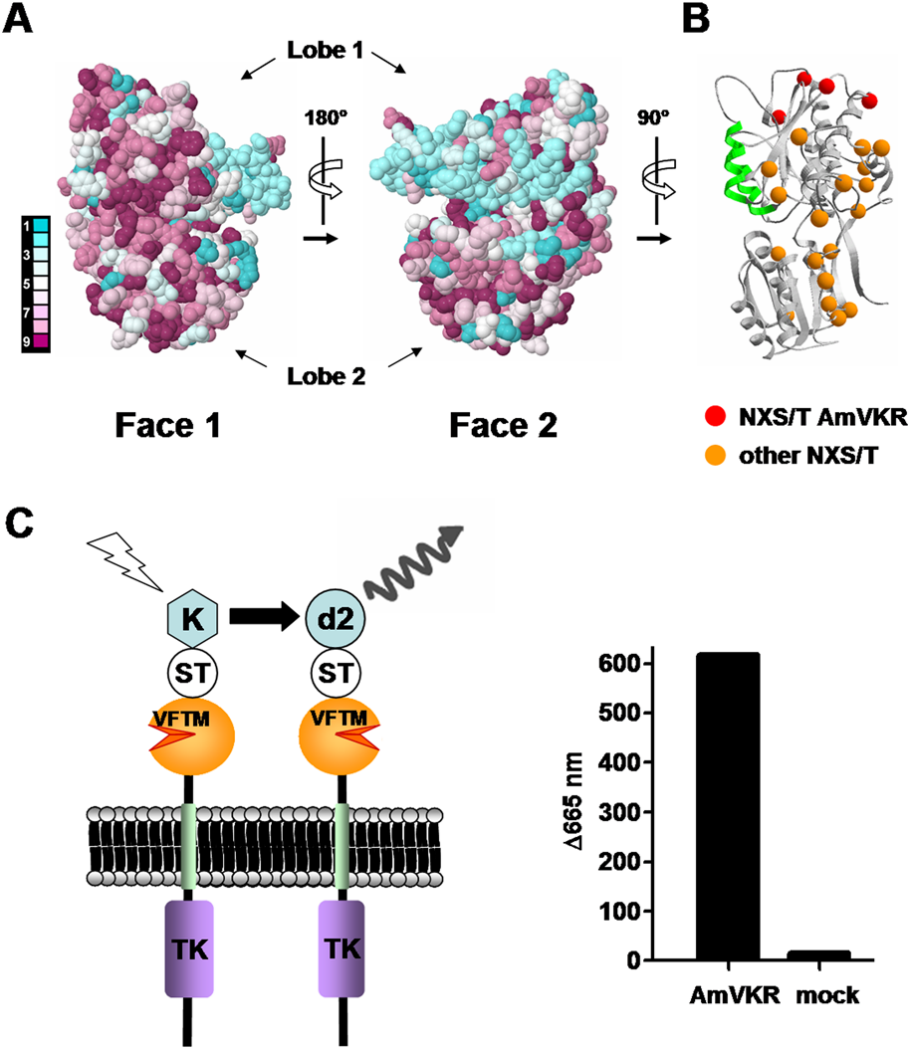
VKR can form dimers. A- Evolutionary conservation of residues at the surface of all VKR VFTMs visualized on both faces of the VFTs (Face 1 and Face 2) using the tridimensional model of AmVKR. Conservation scores are according to a color scale from variable (blue) to conserved (purple) residues. B- Ribbon view of the AmVKR VFTM is shown, with the putative N-glycosylation sites (C-α of Asn residue) found in AmVKR (in red) and in all described VKRs (in orange). C- Time-resolved FRET signal measured between snap-tag (ST) labeled AmVKR subunits at cell surface.

For all RTK, the dimerization is required for the activation, and they also could form large complexes such as homo- and hetero-oligomers [33]. Similarly, it is clearly established that the VFTM of the Class-C GPCRs and ANFR are functioning as dimers [9], [12]. We first performed bioinformatics analyses to predict the dimerization interface of the VKR VFTMs. An evolutionary trace analysis of the surface of VKR VFTMs was performed using a sequence alignment of the VKR VFTMs, and a 3D model of the AmVKR VFTM. This analysis revealed that one face of the subunits is more conserved than the others (Fig. 8A), suggesting that it might correspond to the dimerization interface, as observed with class-C GPCRs and ANFR dimers [9], [34]–[36]. Moreover, none of the putative N-glycosylation sites (consensus sequence Asn-X-Ser/Thr, where X can be any natural amino acid except proline) found in the VKR sequences from different species were located within the proposed dimerization interface (Fig. 8B), as observed with GABA-B receptor dimers [35]. In contrast, most other faces contained at least one putative glycosylation site in at least one of the species examined. Taken together, these observations were consistent with the VFTM dimer interface in the VKRs being similar to that in class C GPCR and ANFR.

Time-resolved FRET measurements using SNAP-tag technology [21] (Fig. 8C) were further performed in order to detect the ability of AmVKR constructs to form dimers at the cell surface. Results in Fig. 8C show that a strong FRET signal was obtained with cells transfected with AmVKR constructs as compared to non transfected cells, indicating that AmVKR proteins effectively constitute dimers at the cell surface.

### VKR can bind amino acids

**Figure 9:**
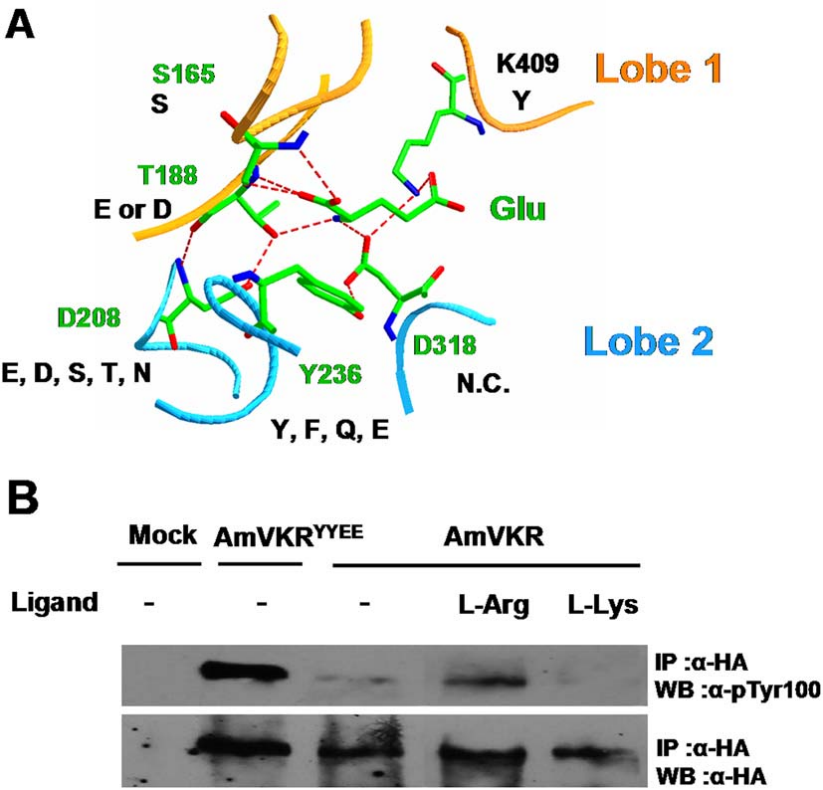
VKR VFTM can bind L-aminoacids responsible for activation. A- Detailed view of glutamate binding in the VFTM of mGlu1 structure. The five residues (labeled in green) responsible for the binding of the α-amino acid functions of the glutamate, and the equivalent residues in VKRs (in black) are shown. The conserved Lys409 in mGlu1 that interact with the carboxylate function of the glutamate side-chain is displayed, and the corresponding tyrosine residue in VKR is indicated. B- Modulation of recombinant AmVKR protein tyrosine kinase activity by aminoacid ligands. Kinase assays were performed on HA-tagged AmVKR immunoprecipitated from transfected HEK293 cells as described in Fig. 7C. L-Arg or L-Lys (100 µM final concentration) amino-acids were added to AmVKR kinase assay reactions, and the constitutively active AmVKR^YYEE^ proteins were used as positive control.

VFTM of most of the class C GPCRs contains the binding site of natural amino acids or derivatives. The amino acid recognition by the VFTM is encoded in an eight residue motif [37] that participates directly or indirectly to the binding of the α-aminoacid functions (primary amine and carboxylic acid). To verify whether the five main residues of the amino acid recognition motif that bind glutamate were conserved in VKRs, we compared the tridimensional model of AmVKR to the structure of mGlu1 VFTM (Fig. 9A). Residue Ser165 that binds the carboxylic acid of the glutamate ligand and that is the most conserved residue in binding sites of Class C GPCR [38], is strictly conserved in VKRs. Other residues that bind the primary amine of the glutamate ligand in mGlu1 (Thr188, Asp208, Tyr236 and Asp318) are less conserved in VKRs, but their chemical features are compatible with the binding of the primary groups of amino-acids. These results suggest that the ligands of VKRs could be amino-acids or derivatives. The strictly conserved residue Lys409 in mGlu1 responsible for the glutamate ligand binding, is replaced by a strictly conserved Tyr in VKRs, indicating that the ligand is probably not glutamate.

VFTM of class C GPCRs binds small ligands [8] like calcium ions (CaSR), glutamate (mGluR), GABA (GABA_B_R), L-Arg and L-Lys (the mammalian GPRC6A [39] and the fish OR5.24 [40]), saccharose or small sugar (sweet taste receptor). We then performed a kinase assay to verify if one of these small molecules could bind and activate AmVKR. Among these molecules, only L-Arg was shown to induce phosphorylation of the receptor (Fig. 9B).

## Discussion

In this study, we reported the discovery of a new family of tyrosine kinase receptors, called VKR, essentially present in invertebrates, and particularly in insects. Using a combination of bioinformatic, molecular biology and biochemical experiments, we demonstrated that the members of this family are single transmembrane-spanning molecules composed of an extracellular VFTM and an intracellular TK domain related to that of IR. At least for *Apis mellifera*, the VKR proteins are functional and their kinase activity can be induced by small ligands such as L-Arg.

The VKR proteins are largely represented in the class of insects, although the first two examples were discovered outside this class, in the trematode *S. mansoni* (the parasite responsible for schistosomiasis, the second most important tropical disease after malaria [41]) [6], and in the sea urchin *S. purpuratus*, and named SmVKR and SpVKR respectively. In 2006, during the inventory of the genes encoding RTK in the *S. purpuratus* genome, two genes Sp-INSR and Sp-ILGFR related to IR family were identified [26], and we could recognize that the second one Sp-ILGFR was an RTK homologous to SmVKR. We could notice that gene structure was overall highly conserved in insects with an important restriction of size and of exon numbers when compared to other organisms. Owing to the substantial number of dipteran *vkr* sequences identified, we could demonstrate that gene structures were homogenous in culicidae (mosquitoes) and drosophilidae (flies). Such a gene conservation in dipterans could argue for a potential importance of VKR receptors in insect biology.

No *vkr* gene could be detected in the lepidopteran genome of *B. mori* and in the the genome of most of the drosophilidae belonging to the *melanogaster* group, despite an extensive and complete analysis of the genome of these flies, suggesting the absence of the *vkr* gene in these insects. In this group, only the *D. ananasse* species was demonstrated to contain a putative *vkr* gene, *Davkr*. Moreover *Davkr* has a genomic structure different from all of the other drosophilidae *vkr* genes with an additional exon in the TK domain that could create an incorrect ATP-binding sequence in the protein and generate an inactive kinase. Such an observation is probably related to the disappearance of *vkr* genes along the evolution of flies inside of the *melanogaster* group. Moreover, for this interpretation, we have to consider that the currently defined *Drosophila* genus has been shown to be paraphyletic, including more than 2 000 species, some but not all of them descending from a common ancestor [42]. Gene mapping and analysis of *vkr* gene environment in the various insects would be helpful for studying evolution of VKR as well as importance in insect biology.

This homogeneity of the VKR proteins in the phylogenetic analyses of VFT and TK domains suggests the existence of a common ancestral gene and the conservation of functional properties in all of these molecules. Although that no information can be given about the origin of *vkr*, we can postulate that, according to the hypothesis already proposed by Yarden and Ullrich about the evolution of RTK [1], an ancestral *vkr* gene would have resulted from the genetic combination of a pre-existing VFTM with a TK domain of a cytoplasmic protein. Until now, *vkr* has never been detected in any vertebrate genome and therefore it can be supposed to be invertebrate specific. However, *vkr* genes have not been found in every invertebrate species and for example are absent from the genome of the nematode *C. elegans*. In platyhelminths, VKR has been characterized only in *Schistosoma* species, but in-depth researches in turbellarian genome data bases have allowed the detection in the planarian *Schmidtea mediterranea* of a genomic sequence (GenBank: AAWT01078636.1) encoding a putative TK domain of VKR proteins linked to a truncated VFT module. Further genomic and functional studies are still required to understand both the origin of *vkr* genes and their selective existence/persistence in given invertebrate organisms.

The VFT domain being close to that of class-C GPCRs in particular GABA_B_ receptors and the TK one being close to that of IR, it suggests an original mode of functioning for a RTK. First, we showed that the recombinant AmVKR forms homo- or oligomers, as expected for membrane receptor containing a VFTM such as class-C GPCRs and ANFR that are well-known to function as homo- or hetero-dimers as well as the RTK [2], [9], [12]. All motifs essential for TK activity were perfectly conserved in the TK domain of VKR suggesting that dimerized AmVKR receptors could autophosphorylate and exert kinase activity. Surprisingly, the basal level of auto-phosphorylation is quite low compared to that we could expect for a recombinant IR in the same conditions [43]. In our study, we need to use constitutively active AmVKR mutants to obtain high levels of kinase activity strongly suggesting that VKR activation in cell signalling was dependent on the binding of a ligand in the extracellular domain of the receptor.

Although all RTKs are activated by dimerization, different ligands employ different strategies for inducing the active dimeric state [33]. VFTMs of membrane receptors are functioning at least as dimers, and they contain the binding site for natural small ligands such as peptides [9], small sugar [44], amino-acids or derivatives [8] and cations [45], [46]. Although the ligand binding site can be at the VFTM dimeric interface [9], [36], usually ligands bind into the VFTM binding pocket and in Class-C GPCRs binding of the natural ligands induces closure of the VFT responsible for receptor activation [12]. In VFTM VKR binding pocket, we observed a relative conservation of the residue responsible for binding of the alpha-amino acid functions of the glutamate in mGlu1, suggesting ligands of VKRs could be amino-acids or derivatives.

Accordingly, among the various molecules known to bind VFTM of class C GPCRs (glutamate, GABA, L-α- amino acids), we could show that L-arginine was able to increase *in vitro* the basal kinase activity of AmVKR, suggesting that this aminoacid could be a preferential ligand for VKR receptors, similarly to the mammalian GPRC6A [39] and the fish OR5.24 [40] receptors, two class C GPCRs for which L-arginine is also a potent agonist. Moreover, it is important to mention that in insects, VFTM-containing mX receptors are modulated by arginine and derivatives [31], [47].

Future investigations will concern the biological role of VKR in development and reproduction, particularly in insects. The presence of high levels of *vkr* transcripts in larval forms and in female gonads of different organisms already supposed the importance of VKR proteins in larval growth and differentiation as well as in reproduction. RNA interference experiments are currently performed in honey bee and mosquitoes in order to elucidate the function of VKR in these processes. Since ovary development in mosquitoes has been shown to be regulated by TOR (Target of Rapamycin)- mediated amino acid signaling [48], [49], we will also investigate the importance of VKR signalling in anautogeny, ie activation of egg development after a blood meal, a unique feature in the life of mosquitoes.

In conclusion, VKR constitute a novel family of RTK specific for invertebrates, mainly expressed in reproductive organs and activated by aminoacids. The biological function of these receptors is yet unknown but if VKR are effectively implied in reproduction of parasites (like schistosomes) or in disease-transmitting insects (like *Anopheles* vector of malaria or *Aedes* vector of filaria and viruses), these receptors already represent interesting drug targets for novel strategies to combat parasitic and infectious diseases.

## Supporting Information

Table S1 Genbank accession numbers of receptors used in phylogenetic analyses.
(0.07 MB PDF)
