# Supplementary material for: A New Family of Receptor Tyrosine Kinases with a Venus Flytrap Binding Domain in Insects and Other Invertebrates Activated by Aminoacids
Source: PLoS One. 2009 May 21;4(5):e5651. doi: 10.1371/journal.pone.0005651 (PMC2680970; doi:10.1371/journal.pone.0005651)
Supplement: Table S1 — Genbank accession numbers of receptors used in phylogenetic analyses. (0.07 MB PDF) [file pone.0005651.s001.pdf]

Table S1

Genbank accession numbers of receptors used in phylogenetic analyses are as follows:

GABABR1 (*A. gambiae*: XP_319474.3, *A. Aegypti*: EAT40934.1, *D. melanogaster*: NP_523569.2, *D. pseudoobscura*: XP_001357356.1, *T. castaneum*: XP_969361.1, *A. mellifera*: XP_392294.3, *N. vitripennis*: XP_001605283.1, *S. purpuratus*: XP_781894.2, *Human*: Q9UBS5, *Mus musculus*: Q9WV18, *Caenorhabditis briggsae*: XP_001675837.1, *Nematostella vectensis*: XP_001639174.1);
GABABR2 (*A. gambiae*: XP_557488.2, *A. Aegypti*: XP_001654541.1, *D. melanogaster*: NP_524438.1, *D. pseudoobscura*: XP_001358144.1, *T. castaneum*: XP_969784.1, *A. mellifera*: XP_393623.3, *S. purpuratus*: XP_782175.2, *Rattus norvegicus*: O88871, *Human*: O75899);
ANFR (*D. melanogaster*: NP_650505.2, *D. pseudoobscura*: XP_001359248.1, *A. gambiae*: XP_307791.4, *A. Aegypti*: XP_001652228.1, *N. vitripennis*: XP_001603765.1, *Bombyx mori*: NP_001036870.1, *Stichopus japonicus*: BAA75224.1, *C. elegans*: AAL77521.1, *Bactrocera dorsalis*: AAM94353.1, *Asterias amurensis*: BAB85468.1, *Hemicentrotus pulcherrimus*: BAA04660.1, *Brissus agassizii*: BAA75197.1, *Diadema setosum*: BAA85332.1, *S. purpuratus*: NP_999705.1)
iGluR (*S. purpuratus*: XP_001178620.1, *Lymnea stagnalis*: AAT40576.1, *C. elegans* : NP_506694.2, *A. gambiae* : XP_314428.4, *A. Aegypti*: XP_001653086.1, *D. melanogaster*: NP_001014714.1, *N. vitripennis*: XP_001606376.1, *A. mellifera*: XP_396271.3, *T. castaneum*: XP_971730.1);
CaSR (*Ciona intestinalis*: ENSCINT00000004255, *Gallus gallus*: XP_416491.1, *Canis familiaris*: XP_545129.2);
Pheromone R (*Human*: XP_946327.1, *M. musculus*: NP_064302.1);
Sweet Taste Receptor (*Human*: Q8TE23, *M. musculus*: Q925I4);
mGluR (*D. melanogaster*: P91685, *T. castaneum*: XP_972407.1, *A. mellifera*: AAR24352.1, *Human*: CAA54796.1, *R. norvegicus*: NP_058708.1, *A. gambiae* : XP_313901.4, *A. Aegypti*: EAT38270.1, *D. pseudoobscura*: XP_001360304.1, *S. purpuratus*: XP_001190166.1)

EGFr (*S. mansoni*: AAA29866.1, *Echinococcus multilocularis*: CAD56486.1, *R. norvegicus* : NP_113695.1, *Human* : CAA25240.1, *C. intestinalis* : NP_001071984.1, *N. vitripennis*: XP_001602830.1, *A. Aegypti*: EAT44302.1, *A. gambiae*: CAC35008.1, *T. castaneum*: XP_971394.1, *D. pseudoobscura*: XP_001361708.1, *D. melanogaster:* AAR85245.1 ,*D. virilis:* ABD64816.1*, S. purpuratus:* SPU-008595 [Urchin genome database])
FGFr (*A. gambiae*: XP_562866.2, *A. Aegypti:* EAT46693.1, *Bombyx mori*: NP_001037558.1 *A. mellifera:* XP_396649.3, *D. pseudoobscura:* XP_001353543.1, *D. melanogaster:* BAA03617.1, *S. purpuratus*: SPU-020677 [Urchin genome database],*T. castaneum:* XP_970831.1, *Spodoptera frugiperda*: BAE94422.1, *Branchiostoma belcheri*: ABD24302.1, *Paracentrotus lividus*: ABG00201.1, *Human*: AAH15035.1, *R. norvegicus* : NP_077060.1
IR *(A. gambiae* : XP_320130.3, *A. Aegypti*: Q93105, *D. melanogaster*: AAC47458, *T. castaneum*: XP_972770, *A. mellifera*: XP_394771, *N. vitripennis*: XP_001606180.1, *S. purpuratus*: ABC61312, *L. stagnalis*: Q25410, *Human* : NP_000199, *M. musculus*: NP_034698, *E. multilocularis* : CAD30260, *S. mansoni* :AAN39120 and AAV65745; C. *elegans* : AAC47715, *B. lanceolatum*: O02466, *Brugia malayi*: XP_001892143, *Biomphalaria glabrata* : AAF31166, *Sycon raphanus* : CAC14729, CAC14730, CAC14731)
RosR (*A. gambiae:* XP_566417.2*, A. Aegypti:* EAT40518.*1, T. castaneum:* XP_970953.1*, D. melanogaster :* CAB55310.1*, D. pseudoobscura :* XP_001354560.1*, Human :* NP_002935.2*, M. musculus :* EDL05074.*1,B. malayi:* EDP29401.1*)*
